# Symptom clusters and core symptoms in early postoperative esophageal cancer patients

**DOI:** 10.3389/fgstr.2026.1766306

**Published:** 2026-09-22

**Authors:** Menglan Zhong, Changhong Wang, Xiaoqi Liu, Yan Wang, Xiaona Qi, Shaohua Xie

**Affiliations:** 1 Department of Nursing, Harbin Medical University Cancer Hospital, Harbin Medical University, Harbin, Heilongjiang, China; 2 School of Public Health, Fujian Medical University, Fuzhou, China; 3 Upper Gastrointestinal Surgery, Department of Molecular Medicine and Surgery, Karolinska Institutet, Stockholm, Sweden

**Keywords:** cluster analysis, esophageal neoplasms, network analysis, postoperative care, symptom assessment

## Abstract

**Purpose:**

Patients undergoing esophagectomy frequently experience diverse and complex symptoms. This study aimed to identify clusters of early postoperative symptoms and determine core symptoms among these patients, to facilitate targeted symptom management strategies.

**Methods:**

This prospective cohort study included 361 patients who underwent esophageal cancer surgery at a tertiary Grade A hospital in Harbin between 2023 and 2024. The European Organisation for Research and Treatment of Cancer Quality of Life Questionnaire Core 30 (QLQ-C30) and the Esophageal Cancer-Specific Module (QLQ-OES18) were used to assess 18 symptoms one month postoperatively. Exploratory factor analysis and network analysis were conducted to identify symptom clusters and core symptoms, respectively. A 1-month landmark survival analysis was performed to examine their associations with 1-year postoperative survival, using a parsimonious multivariable Cox regression model.

**Results:**

Five distinct symptom clusters were identified: eating-related (eating difficulty, reflux, choking, appetite loss, nausea, vomiting, dysphagia; prevalence 49.0%), breathing-related (cough, dyspnea, insomnia; prevalence 47.0%), pain-related (esophageal pain, generalized pain; prevalence 48.0%), fatigue-speech related (fatigue, dry mouth, trouble talking; prevalence 51.0%), and excretion-related (constipation, diarrhea; prevalence 50.0%). Network analysis identified fatigue (relative strength [rS] = 2.83), pain (rS = 1.69), and insomnia (rS = 1.58) as core symptoms, with fatigue serving as the key bridge symptom. In the 1-month landmark analysis, no statistically significant association was detected between symptom clusters or core symptoms and 1-year postoperative survival.

**Conclusion:**

This study identified distinct symptom clusters and core symptoms one month post-esophagectomy that merit clinical attention during postoperative follow-up. Specifically, fatigue represents a key therapeutic target for developing intervention strategies.

## 1 Introduction

Esophageal cancer is the seventh leading cause of cancer-related deaths globally (1). China bears a disproportionate burden, accounting for approximately 50.0% of global cases (2). Surgical resection, typically combined with therapies such as chemotherapy and radiotherapy, is the primary curative treatment. However, as the primary therapeutic intervention, surgery frequently results in a constellation of multidimensional symptoms, predominantly due to anatomical changes in the digestive tract.

Postoperative symptoms are generally more severe and complex in the early phase. The first postoperative month represents a critical transition period, as patients progress from enteral to oral nutrition, during which symptom exacerbation is documented. Current estimates indicate that over 50.0% of patients experience complications at this stage, with serious complications such as pulmonary infections and anastomotic leaks being common (3, 4). These complications impede recovery and are associated with increased mortality. Furthermore, patients frequently experience psychological distress during this phase, including fear of recurrence, uncertainty about outcomes, and impaired daily function (5).

Postoperative patients frequently present with multiple coexisting symptoms forming interactive clusters. These clusters typically comprise two or more concurrent symptoms sharing a common etiology or pathological mechanism (6). Previous studies have identified symptom clusters following esophagectomy, including gastrointestinal, dysphagia-related, fatigue, and pain clusters (7). However, conventional statistical methods used in prior research have addressed complex symptom interactions only to a limited extent. Network analysis, an emerging computational modeling framework, provides a means to understand these complex inter-symptom relationships (8). Originally applied in psychopathology to investigate symptom interactions (9), network analysis has recently been extended to cancer research. This methodology identifies core symptoms reliably by mapping symptom network topology and quantifying centrality measures, thereby establishing a theoretical foundation for developing targeted interventions.

Research on early post-esophagectomy symptoms using network and exploratory factor analysis remains limited, with most studies focusing on the first postoperative week or mixed treatment phases while neglecting the critical one-month recovery period. Existing approaches have also failed to help these profiles’ survival. This study combined both methods to identify symptom clusters and core symptoms after esophagectomy and examined their associations with one-year postoperative mortality. This study combined both methods to identify symptom clusters and core symptoms after esophagectomy, and examined their associations with one-year postoperative mortality using a 1-month landmark design to avoid immortal time bias.

## 2 Methods

### 2.1 Study design and participants

This prospective cohort study was conducted at a tertiary Grade A hospital in Harbin, China, from April 7, 2023, to March 4, 2024. A total of 378 consecutive patients who underwent curative-intent esophagectomy for esophageal cancer were initially enrolled.

Inclusion criteria were (1): age ≥18 years; (2) histologically confirmed primary esophageal cancer scheduled for initial curative-intent esophagectomy; (3) provision of written informed consent; (4) intact cognitive function for independent questionnaire completion.

Twelve patients (3.0%) were lost to follow-up before the 1-month symptom assessment, and five (1.0%) died within 30 days postoperatively. Both groups did not complete the scheduled symptom evaluation, so they were excluded from all symptom analyses and the subsequent 1-month landmark survival analysis. The final analytic cohort for symptom assessment included 361 participants.

All patients were followed postoperatively, with symptom assessments conducted at 1 month after surgery. For the landmark survival analysis, follow-up time was counted from the 1-month landmark time point (postoperative day 31) to 1 year postoperatively. This study was conducted in accordance with the Declaration of Helsinki and approved by the Ethics Committee of a tertiary Grade A hospital in Harbin on February 14, 2023 (No. KY20XX-XX).

### 2.2 EORTC QLQ-C30

This study employed the widely validated 30-item EORTC QLQ-C30 (version 3.0) to assess health-related quality of life in cancer patients (10). To comprehensively capture both general cancer-related symptoms and those specific to esophageal cancer, our analysis focused specifically on its symptom assessment module, which includes three multi-item subscales—fatigue, pain, and nausea/vomiting—and six single-item symptom measures: dyspnea, insomnia, appetite loss, constipation, diarrhea, and financial difficulties. The Chinese version of the scale was used in this investigation, with established reliability documented in prior validation studies. Cronbach’s α coefficients for the relevant symptom subscales have been consistently reported in the range of 0.70–0.80 (11), reflecting satisfactory measurement reliability.

### 2.3 EORTC QLQ-OES18

The QLQ-OES18 is an 18-item module developed to evaluate disease-specific concerns related to esophageal cancer and its treatment in patients (12). It comprises four multi-item symptom scales (dysphagia, eating difficulties, reflux, and esophageal pain) and six single-item symptoms (difficulty swallowing saliva, choking when swallowing, dry mouth, taste problems, cough, and speech difficulties). The Chinese version of the QLQ-OES18 shows satisfactory internal consistency, with Cronbach’s α for its symptom subscales generally 0.689–0.822 (13).

All items in the two questionnaires—except for the global health status item of the QLQ-C30—were rated on a 4-point Likert scale (1 = “Not at all” to 4 = “Very much”). Scale raw scores were derived by averaging the corresponding items. Following linear transformation to a 0–100 standardized scale, higher scores on symptom scales reflect more severe symptom burden. In accordance with established methodology in oncology quality-of-life research (14, 15), symptom scores were dichotomized to identify clinically significant burden. For each symptom scale, raw scores were computed following the EORTC QLQ scoring manual. A binary variable was then created using a clinically defined cutoff: a symptom was considered present if any item within the scale was rated 3 (“Quite a bit”) or 4 (“Very much”), and absent if all items were rated 1 (“Not at all”) or 2 (“A little”). This threshold corresponds to symptom severity levels that typically warrant clinical attention, thereby enhancing the practical relevance of the identified symptom clusters. Although dichotomization reduces statistical granularity, it was selected to ensure a clear definition of clinically meaningful symptom groups and to maintain consistency with key comparative studies.

### 2.4 Socio-demographic and clinical data

Socio-demographic and clinical variables included: age (<60, 60–74, or ≥75 years) (15), pathological stage (0–I, II, or III–IV), tumor location (upper/mid/lower thoracic esophagus or gastroesophageal junction), surgical approach (Ivor Lewis, McKeown), number of incisions (1, 2, or ≥3), and receipt of Neoadjuvant therapy (yes/no). Postoperative complication count (0, 1, or >1) and comorbidity burden (0 or ≥1) were documented. All data were extracted from medical records by a senior surgeon.

Postoperative complications were predefined and included perioperative hemorrhage; anastomotic leakage, perforation, or stenosis; wound infection requiring intervention or dehiscence; gastric perforation; intra-abdominal or intrathoracic abscess; intestinal obstruction; thoracic duct injury; reintubation or prolonged mechanical ventilation; recurrent laryngeal nerve palsy; pneumonia; pulmonary embolism; and myocardial infarction. Comorbidity included angina pectoris, heart failure, hypertension, diabetes mellitus, pulmonary disease, renal insufficiency, and hepatic failure.

### 2.5 Statistical analysis

All statistical analyses were performed using R software, except for the exploratory factor analysis (EFA), which was conducted using SPSS. Symptom clustering employed a two-stage approach. First, EFA identified clusters using principal axis factoring with Varimax rotation. Symptoms with factor loadings ≥.40 were retained; those cross-loading (≥.40 on multiple factors) were assigned to the factor with their highest loading. Factor retention was guided by Kaiser’s criterion (eigenvalue >1), parallel analysis, and scree plot evaluation. Data suitability was confirmed via the Kaiser-Meyer-Olkin (KMO) measure and Bartlett’s test of sphericity. The EFA was performed using standardized 0–100 symptom severity scores.

To explore robustness across different methodological frameworks, we additionally applied principal component analysis (PCA; eigenvalue cutoff=1) and hierarchical cluster analysis (HCA; minimum 1-R² ratio) as complementary exploratory techniques. PCA and EFA address fundamentally different statistical objectives: PCA maximizes total variance explained through linear combinations of observed variables, whereas EFA identifies latent constructs that explain covariance among symptoms. Therefore, PCA and HCA results were used as exploratory references rather than confirmatory evidence, with the final cluster solution selected based on clinical interpretability and theoretical coherence.

Seventeen symptoms with ≥10.0% prevalence were analyzed: eating difficulty, dysphagia, reflux, choking when swallowing, appetite loss, nausea/vomiting, cough, dyspnea, insomnia, esophageal pain, pain, fatigue, dry mouth, trouble talking, trouble swallowing saliva, constipation, and diarrhea.

Second-stage cluster membership was defined based on the number of manifested symptoms within each factor-derived cluster. To align with the conceptual definition of symptom clusters — co-occurrence of at least two concurrent symptoms — a two-tiered threshold was applied: for clusters containing ≥3 symptoms, presence was defined as manifestation of ≥50% of symptoms (inherently requiring ≥2 symptoms); for clusters containing exactly 2 symptoms, presence required co-occurrence of both symptoms (16). Patients could be assigned to multiple clusters.

Within-cluster differences in baseline characteristics were examined using chi-square tests. Multivariable logistic regression identified factors associated with each symptom cluster, adjusting for age, sex, pathological stage, surgical procedure, neoadjuvant therapy, postoperative complications, and comorbidities. Results are presented as adjusted odds ratios (ORs) with 95% CIs.

A Mixed Graphical Model (MGM) was employed to estimate the symptom network using the mgm package. The network included 16 symptom nodes specified as continuous variables (0–100 severity scores). Covariates (surgical method, postoperative hospitalization duration, and neoadjuvant therapy) were included as control variables. The model order was set to 2 (pairwise interactions), with regularization applied via graphical LASSO (λ selected by 10-fold cross-validation) and model selection using EBIC (γ = 0.25). Edges were retained only if selected by LASSO. Covariates(surgical method, postoperative hospitalization duration, and preoperative neoadjuvant therapy) were included. These variables were selected based on preliminary evidence of association with the outcome (p < 0.05). Their inclusion allowed for statistical adjustment of their effects, thereby strengthening the validity of the estimated relationships between primary variables (17). Edges represent regularized partial correlation coefficients; positive edges indicate positive associations, negative edges indicate inverse associations. Node predictability (R²) was estimated via the MGM package (18).

Centrality measures (Strength, Closeness, Betweenness, Expected Influence) (19)were calculated using qgraph. Strength was selected as the primary indicator for core symptoms based on its superior stability and reliability (20, 21). Relative strength (rS) was defined as the standardized Strength value (Z-score). Bridge centrality was assessed using networktools, with bridge strength calculated as the sum of edge weights from a node to all nodes in different communities (defined by the five EFA-derived clusters). Network stability was evaluated via bootnet (1000 bootstrap samples). Correlation Stability (CS) coefficients were calculated separately for each centrality index (CS >0.25 acceptable, >0.50 good). Bootstrap centrality difference tests (1000 samples) were performed for Strength centrality. Edge weight accuracy was assessed using bootstrap-derived 95% CIs.

A 1-month landmark analysis was applied to avoid immortal time bias, with postoperative day 30 set as the landmark. Only patients alive at this landmark and with symptom data were included. Follow-up was counted from day 31; the primary endpoint was 1-year all-cause mortality. Patients alive, lost to follow-up, or withdrawn were right-censored at last known survival status. Kaplan-Meier curves and log-rank tests compared survival between groups. The proportional hazards assumption was verified using Schoenfeld residual tests (P >0.05). Univariable Cox models estimated crude hazard ratios (HRs) with 95% CIs.

## 3 Results

### 3.1 Participants

This study initially recruited 378 patients. Twelve patients (3.0%) were lost to follow-up, and five (1.0%) died within one month postoperatively, resulting in a final cohort of 361 participants.

The cohort comprised predominantly male patients (96.7%), aged 60–74 years (53.7%), with stage 0-I tumors (47.4%). Most underwent laparoscopic surgery (56.5%). Neoadjuvant therapy was administered to 46.5% of patients. Postoperative complications occurred in 30 patients (8.3%), and 13.6% presented with ≥1 comorbidity (**Table 1**).

**Table 1:** Distribution of baseline characteristics of 361 patients undergoing esophageal cancer surgery by presence of symptom clusters, number (%).

Characteristics	Total	Symptom clusters									
		Eating-related		Breathing-related		Pain-related		Fatigue-speech related		Excretory-related	
		Present (n=178)	Absent (n=183)	Present (n=170)	Absent (n=191)	Present (n=174)	Absent (n=187)	Present (n=184)	Absent (n=177)	Present (n=182)	Absent (n=179)
Sex											
Male	349 (96.7)	171 (96.1)	178 (97.3)	166 (97.6)	183 (95.8)	167 (96.0)	182 (97.3)	179 (97.3)	170 (96.0)	175 (96.2)	174 (97.2)
Female	12 (3.3)	7 (3.9)	5 (2.7)	4 (2.4)	8 (4.2)	7 (4.0)	5 (2.7)	5 (2.7)	7 (4.0)	7 (3.8)	5 (2.8)
Age											
<60	158 (43.8)	81 (45.5)	77 (42.1)	75 (44.1)	83 (43.5)	78 (44.8)	80 (42.8)	81 (44.0)	77 (43.5)	68 (37.4)	90 (50.3)
60-74	194 (53.7)	92 (51.7)	102 (55.7)	91 (53.5)	103 (53.9)	90 (51.7)	104 (55.6)	99 (53.8)	95 (53.7)	109 (59.9)	85 (47.5)
>74	9 (2.5)	5 (2.8)	4 (2.2)	4 (2.3)	5 (2.6)	6 (3.0)	3 (1.6)	4 (2.2)	5 (2.8)	5 (2.7)	4 (2.2)
Pathological stage											
0-I	171 (47.4)	87 (48.9)	84 (45.9)	80 (47.1)	91 (47.6)	83 (47.7)	88 (47.1)	86 (46.7)	85 (48.0)	75 (41.2)	96 (53.6)
II	77 (21.3)	39 (21.9)	38 (20.8)	38 (22.4)	39 (20.4)	41 (23.6)	36 (19.3)	44 (23.9)	33 (18.6)	43 (23.6)	34 (19.0)
III-IV	113 (31.3)	52 (29.2)	61 (33.3)	52 (30.6)	61 (31.9)	50 (28.7)	63 (33.7)	54 (29.3)	59 (33.3)	64 (35.2)	49 (27.4)
Histology											
Squamous cell carcinoma	336 (93.1)	165 (92.7)	171 (93.4)	158 (92.9)	178 (93.2)	162 (93.1)	174 (93.0)	171 (92.9)	165 (93.2)	169 (92.9)	167 (93.3)
Adenocarcinoma	12 (3.3)	6 (3.4)	6 (3.3)	6 (3.5)	6 (3.1)	6 (3.4)	6 (3.2)	6 (3.3)	6 (3.4)	6 (3.3)	6 (3.3)
Unspecified	13 (3.6)	7 (3.9)	6 (3.3)	6 (3.5)	7 (3.7)	6 (3.4)	7 (3.7)	7 (3.8)	6 (3.4)	7 (3.8)	6 (3.4)
Surgical approach											
Ivor Lewis	49 (13.6)	24 (13.5)	25 (13.7)	23 (13.5)	26 (13.6)	24 (13.8)	25 (13.4)	25 (13.6)	24 (13.6)	25 (13.7)	24 (13.4)
McKeown	312 (86.4)	154 (86.5)	158 (86.3)	147 (86.5)	165 (86.4)	150 (86.2)	162 (86.6)	159 (86.4)	153 (86.4)	157 (86.3)	155 (86.6)
Neoadjuvant therapy											
Yes	168 (46.5)	84 (47.2)	84 (45.9)	78 (45.9)	90 (47.1)	82 (47.1)	86 (46.0)	84 (45.7)	84 (47.5)	73 (40.1)	95 (53.1)
No	193 (53.5)	94 (52.8)	99 (54.1)	92 (54.1)	101 (52.9)	92 (52.9)	101 (54.0)	100 (54.3)	93 (52.5)	109 (59.9)	84 (46.9)
Postoperative complications											
No	331 (91.7)	164 (92.1)	167 (91.3)	154 (90.6)	177 (92.7)	163 (93.7)	168 (89.8)	169 (91.8)	162 (91.5)	163 (89.6)	168 (93.9)
Yes	30 (8.3)	14 (7.9)	16 (8.7)	16 (9.4)	14 (7.3)	11 (6.3)	19 (10.2)	15 (8.2)	15 (8.5)	19 (10.4)	11 (6.1)
Comorbidities											
0	312 (86.4)	153 (86.0)	159 (86.9)	144 (84.7)	168 (88.0)	155 (89.1)	157 (84.0)	158 (85.9)	154 (87.0)	150 (82.4)	162 (90.5)
≥1	49 (13.6)	25 (14.0)	24 (13.1)	26 (15.3)	23 (12.0)	19 (10.9)	30 (16.0)	26 (14.1)	23 (13.0)	32 (17.6)	17 (9.5)

Among the 361 patients who completed the 1-month postoperative symptom assessment, 293 patients with complete 1-year follow-up survival data were included in the primary 1-month landmark survival analysis. The remaining 68 patients were excluded from the primary complete-case analysis for the following reasons: 42 were lost to follow-up after the 1-month symptom assessment, 18 voluntarily withdrew from the study, and 8 had incomplete survival outcome records. In sensitivity analyses, all 68 patients were treated as right-censored at their last known follow-up date to maximize utilization of available time-to-event data.

### 3.2 Symptom clusters

To ensure analytical validity, only symptoms with incidence ≥10.0% were included in cluster analysis (22, 23), as low-prevalence symptoms may compromise results. The symptom “difficulty swallowing saliva,” identified as a single-item factor in exploratory factor analysis (EFA), was excluded since clusters require ≥2 symptoms. Consequently, 16 symptoms were analyzed using principal axis factoring with Varimax rotation. EFA feasibility was confirmed (Kaiser-Meyer-Olkin measure = .70; Bartlett’s test of sphericity: χ² = 754.50, *P* <.001). The analysis yielded five factors accounting for 52.2% cumulative variance. Based on symptoms comprising each factor, clusters were labeled: (1) eating-related, (2) breathing-related, (3) pain-related, (4) fatigue-speech-related, and (5) excretion-related. (**Supplementary Table S1**).

To mitigate methodological dependency and cross-validate the exploratory factor analysis (EFA), principal component analysis (PCA), and hierarchical cluster analysis (HCA) were employed. PCA yielded two components, but the resulting clusters were overly broad and failed to capture the detailed symptom associations. The five clusters produced by HCA were marked by considerable symptom overlap and poorly defined boundaries, thereby compromising clinical interpretability. Comparatively, the five-factor structure from EFA exhibited clearer distinctions between categories, closer consistency with theoretical frameworks and clinical observations, and enhanced structural validity. Consequently, the EFA five-cluster solution was retained for further analysis. Furthermore, we identified two high-risk subgroups for postoperative fatigue: patients aged 60-74 years (Adjusted OR = 1.84, 95% CI: 1.06-3.23) and those receiving neoadjuvant therapy (Adjusted OR = 1.86, 95% CI: 1.05-3.35). (**Supplementary Table S2**).

### 3.3 Network analysis

Using MGM for the 16 continuous symptom nodes conditioned on the three covariates (surgical method, postoperative hospitalization duration, and neoadjuvant therapy). Strongest conditional dependencies were observed between esophageal pain and generalized pain, eating difficulty and choking when swallowing, and nausea/vomiting and reflux (**Figure 1**), ranging from 4.0% to 36.9%. The high predictability of fatigue (FA, 36.9%) indicates that over one-third of its variance is explained by neighboring symptoms. Centrality analysis identified fatigue (rS = 2.83), pain (rS = 1.69), and insomnia (rS = 1.58) as having the highest strength centrality. Constipation exhibited the lowest strength centrality (rS = .42; **Supplementary Figures S1**). Fatigue also demonstrated the highest bridge strength (rBS = 1.09; **Supplementary Figure 2**). Network stability assessment for strength and expected influence centrality yielded a mean Correlation Stability (CS) coefficient of.52 (CS >.50 indicates good stability). Bootstrap analysis confirmed high edge weight accuracy, with narrow 95% confidence intervals closely aligning with estimated weights (**Figures 2**).

**Figure 1:**
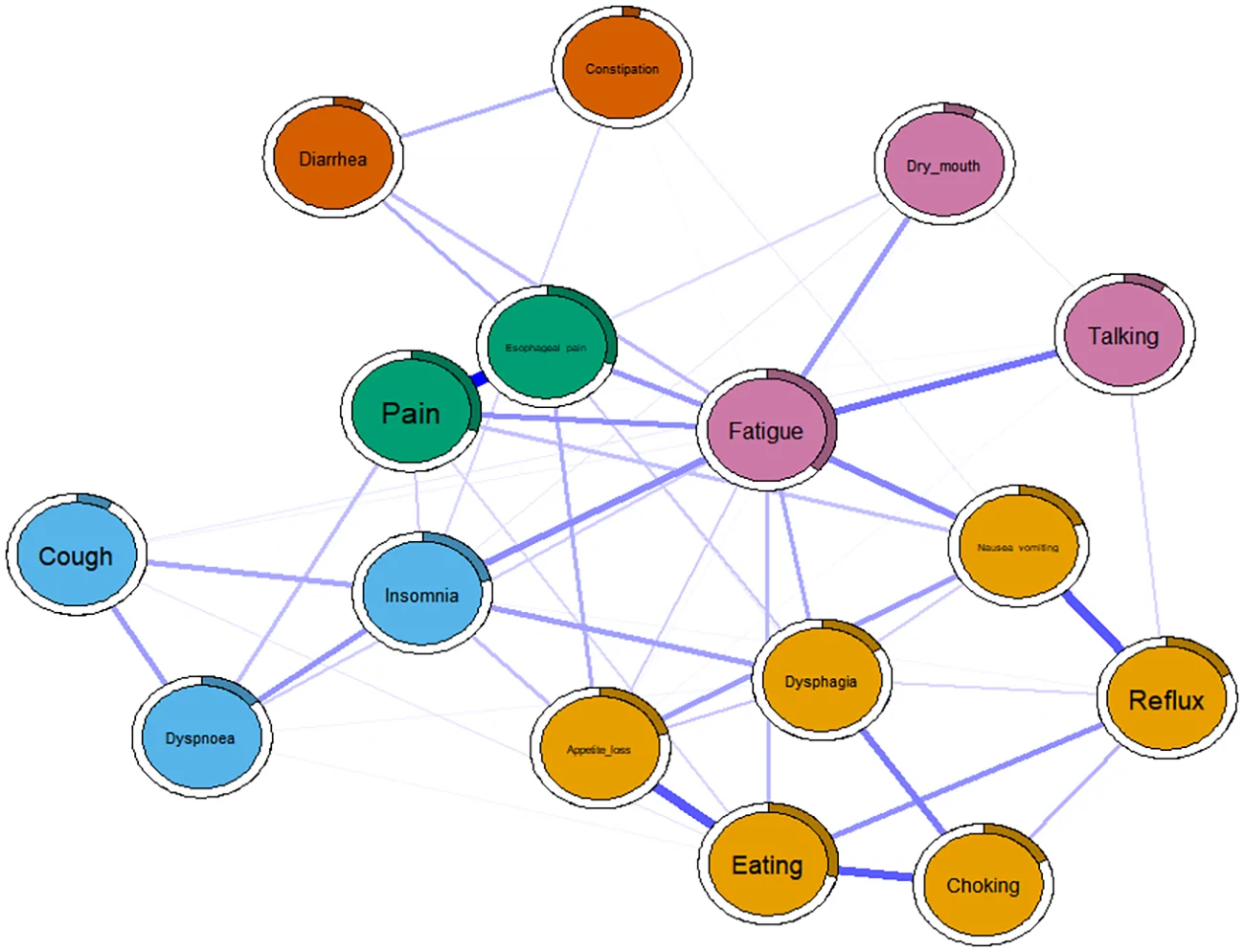
Visualization of symptom network one month after esophageal cancer surgery, including Constipation, Diarrhoea, Drymouth, Talking, Fatigue,Pain, Esophageal pain, Fatigue, Insomnia, Cough, Dyspnoea, Appetiteloss, Dysphagia, Eating, Choking, Reflux, Nausea and vomiting,Color-coded nodes represent symptom categories, while line thickness indicates the strength of associations.

**Figure 2:**
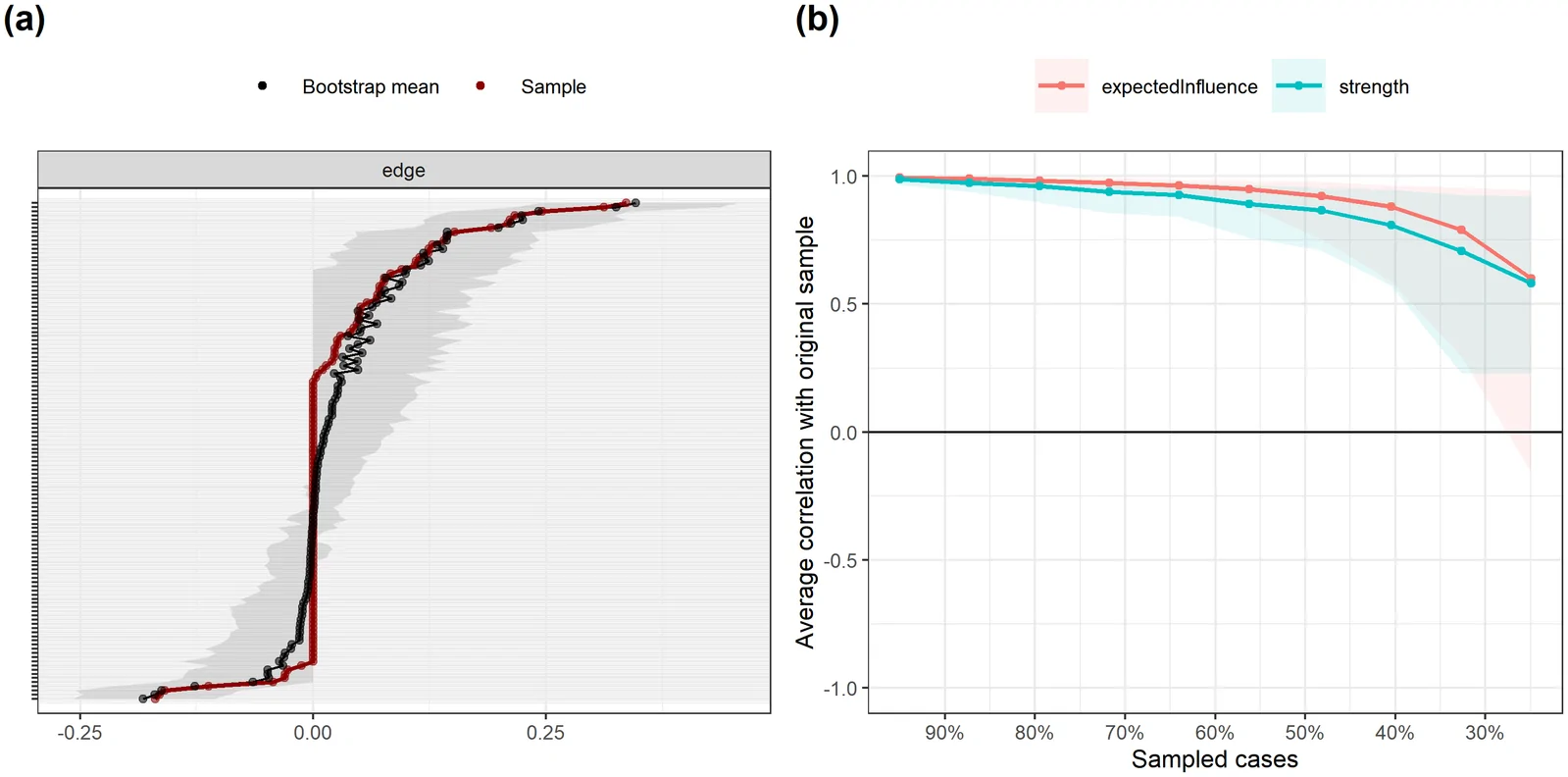
Reliability assessment of symptom networks: **(A)** Network stability coefficients **(B)** Edge weight accuracy via bootstrap analysis. Panel **(A)** presents a line plot comparing bootstrap means (black) and sample values (red) for edge estimates with confidence intervals. Panel **(B)** displays a line chart of average correlations, comparing expected influence (red) and strength (blue) with shaded confidence intervals.

### 3.4 Symptoms and 1-year survival

In the primary 1-month landmark analysis (n = 293), the median follow-up duration from the landmark time point was 335 days (interquartile range: 312-358 days). A total of 16 deaths (5.5%) occurred during the follow-up period. Schoenfeld residual tests confirmed that all variables in all models satisfied the proportional hazards assumption (all *P* > 0.05).

Kaplan-Meier analysis showed no statistically significant differences in 1-year survival rates between patients with or without any symptom cluster or core symptom (all log-rank *P* > 0.05). In univariable Cox regression, none of the symptom clusters or core symptoms were significantly associated with increased 1-year mortality risk (**Table 2**). After adjustment for pathological stage and postoperative complications in the parsimonious multivariable model, no statistically significant associations were detected between any symptom cluster or core symptom and 1-year mortality (**Table 2**).

**Table 2:** Associations of symptom clusters and core symptoms with 1-year survival after esophageal cancer surgery, expressed as hazard ratios (HR) with 95% confidence intervals (CI)(N=293).

Symptoms	Number of patients	Number of deaths (%)	Crude HR (95% CI)
Eating-related cluster			
No	149	5 (3.4)	1.00 (reference)
Yes	144	11 (7.6)	1.90 (0.64-5.67)
Breathing-related cluster			
No	158	7 (4.4)	1.00 (reference)
Yes	135	9 (6.7)	1.20 (0.42-3.42)
Pain-related cluster			
No	147	5 (3.4)	1.00 (reference)
Yes	146	11 (7.5)	1.82 (0.61-5.43)
Fatigue-speech-related cluster			
No	143	7 (4.9)	1.00 (reference)
Yes	150	9 (6.0)	1.19 (0.44-3.21)
Excretory-related cluster			
No	151	6 (4.0)	1.00 (reference)
Yes	142	10 (7.0)	1.79 (0.65-4.94)
Fatigue			
No	74	3 (4.1)	1.00 (reference)
Yes	219	13 (5.9)	1.25 (0.35-4.50)
Pain			
No	173	10 (5.8)	1.00 (reference)
Yes	120	6 (5.0)	0.57 (0.18-1.81)
Insomnia			
No	233	14 (6.0)	1.00 (reference)
Yes	60	2 (3.3)	0.46 (0.10-2.06)

(Corrected covariates: Sex, Age, Pathological stage, Surgical approach, Neoadjuvant therapy, Postoperative complications, and Comorbidities).

Given the limited number of mortality events and wide 95% confidence intervals of effect estimates, the statistical power of this study was insufficient to detect small-to-moderate survival differences. The null finding requires further validation in larger multicenter cohorts.

## 4 Discussion

Exploratory factor analysis identified five symptom clusters at 1-month post-esophagectomy: eating-related, respiratory-related, pain-related, fatigue-speech-related, and excretion-related clusters. Mixed Gaussian Graphical Model (MGM) network analysis revealed fatigue, pain, and insomnia as core symptoms, with fatigue demonstrating pivotal bridging functionality. However, neither symptom clusters nor core symptoms were associated with 1-year postoperative survival. These findings are hypothesis-generating and suggest that targeting central symptoms—particularly fatigue due to its bridging role—may potentially reduce overall symptom burden through network effects. However, given the cross-sectional nature of our network analysis, these hypotheses require prospective validation in future interventional or longitudinal studies.

### 4.1 Symptomatic experiences

This study observed a prevalent multisymptom burden among esophagectomy patients at one month postoperatively. Dysphagia (93.1%; severity score=22.33) and fatigue (74.8%; severity score=33.33) demonstrated the highest frequency and severity, consistent with existing evidence (24, 25). Dysphagia, a frequent complication during early recovery that may persist for years, arises from multifactorial origins, including surgical trauma-induced tissue edema, vagal nerve injury causing esophageal dyskinesia, and impaired swallowing muscle coordination (26, 27). This symptom adversely affects cancer patients’ quality of life (28). As the predominant somatic symptom, fatigue is significantly associated with impaired health-related quality of life and reduced cancer-specific survival (29). Furthermore, reflux and eating difficulties are prevalent during this phase, with their manifestation closely linked to surgical factors and postoperative recovery processes. Although existing studies (7, 30) identified depression and anxiety as predominant conditions at one month post-surgery, with common concurrent symptoms including nausea/vomiting, dyspnea, fatigue, pain, appetite loss, dysphagia, and dysgeusia. These discrepancies may arise from methodological differences in symptom assessment across studies.

### 4.2 Systematic classification of symptom clusters

This study identified five symptom clusters in 361 esophagectomy patients at one month postoperatively: Eating-Related (eating difficulties, reflux, choking when swallowing, appetite loss, nausea/vomiting, dysphagia; 49.0%), Breathing-Related (cough, dyspnea, insomnia; 47.0%), Pain-Related (esophageal pain, pain; 48.0%), Fatigue-speech related (fatigue, dry mouth, trouble talking; 51.0%), and Excretory-Related (constipation/diarrhea; 50.0%). Approximately 67.0% of patients exhibited two or more concurrent clusters, indicating a significant comorbid symptom burden consistent with established literature (31). Although symptom clustering analysis offers only preliminary identification of cancer-related symptoms with shared features, existing evidence reveals heterogeneity in this study’s symptom cluster composition. Among symptom clusters largely aligned with prior research, fatigue is seldom categorized alongside dry mouth and dysarthria. Clinically, dry mouth and dysarthria frequently co-occur during and following chemoradiation (32, 33), attributable to shared pathophysiological mechanisms—predominantly salivary gland impairment and cranial nerve damage. Given that radiotherapy directly exacerbates fatigue, clustering these symptoms is clinically justified. This cluster exhibited the highest incidence (51.0%) in our cohort, necessitating dedicated management protocols. Methodologically, discrepancies in cluster composition likely originate from multifactorial confounders (e.g., patient characteristics, study design, assessment tools, statistical methods), complicating clinical interpretation. Nevertheless, our findings establish the prevalence and clinical significance of this symptom complex, warranting prioritized investigation into its underlying mechanisms and management strategies to alleviate patient burden.

### 4.3 Core symptoms

This study documented a fatigue prevalence of 74.3% among post-esophagectomy patients, with a mean severity score of 33.33. Fatigue exhibited the highest centrality metrics within the symptom network (strength [rS] = 2.83; bridge strength [rBS] = 1.09), confirming its dual role as both a core postoperative symptom at one month and a pivotal bridge node interconnecting symptom clusters. These findings align with Zhu (7) et al. and extend them by elucidating fatigue’s specific bridging properties. In contrast, Chen (32) et al. identified gastroesophageal reflux, rather than fatigue, as the predominant symptom. This discrepancy likely reflects methodological variations, including differences in sample characteristics and assessment tools. Critically, 66.0% of patients did not report fatigue symptoms to clinicians, and 43.0% lacked awareness of management strategies (34). Despite this underreporting and knowledge gap, fatigue imposes a substantial burden, persistently impairing health-related quality of life (HRQL) in this population (35). These factors exacerbate physical and psychological dysfunction, increase healthcare utilization, and worsen prognosis. Targeted interventions addressing core and bridge symptoms (e.g., fatigue) may represent a promising approach to mitigating cascade effects among symptom clusters, potentially enhancing precision in personalized symptom management. However, this hypothesis needs to be tested in future interventional studies. According to the NCCN guidelines (36), a recommended four-phase management paradigm—comprising screening, assessment, intervention, and tracking—should be integrated, specifically incorporating personalized exercise programs.

This investigation further identified pain (strength centrality [rS] = 1.69) and insomnia (rS = 1.58) as key central symptoms. Evidence indicates (37) that 30.0%–50.0% of patients experience acute or chronic post-esophagectomy pain, consistent with our findings. Persistent pain significantly impedes respiratory function recovery and overall quality of life, with effective postoperative pain management being a critical determinant of early quality-of-life outcomes following esophagectomy (38). Despite extensive research on pain in esophageal cancer, quantification of its symptom centrality via network analysis remains limited, potentially hindering targeted management strategies. A centrality-driven symptom prioritization model could advance beyond conventional experience-based approaches, enabling more precise symptom management. Insomnia, also identified as a significant central symptom (rS = 1.64), arises from multiple interacting pathogenic factors. Patients experience adverse effects due to diverse postoperative physical, psychological, and social challenges, particularly during the initial recovery phase.

Furthermore, insomnia functioned as a secondary bridge node within the symptom network (bridge strength [rBS] = .55). Existing evidence (39) indicates that postoperative sleep disturbances may persist for up to 90 days, with peak severity occurring during early recovery. Clinical observations reveal significantly elevated psychological stress during this period, establishing a bidirectional exacerbating relationship between sleep disturbances and psychological distress. Zhang (40) et al. documented persistent insomnia exceeding 12 months post-esophagectomy. This study elucidates that despite potentially lower prevalence and intensity, insomnia acts as both a central symptom and a pivotal cross-dimensional bridge. This finding suggests clinicians may underestimate its topological significance in current practice, potentially contributing to delayed recognition and suboptimal management. Evidence from a systematic review (41) supports a comprehensive insomnia management approach comprising (1): screening and assessment, (2) non-pharmacological interventions (e.g., sleep hygiene education, cognitive behavioral therapy for insomnia [CBT-I], mindfulness-based stress reduction), and (3) judicious pharmacological treatments (e.g., melatonin, zolpidem, zopiclone, or dual orexin receptor antagonists). Clinicians should employ standardized instruments for insomnia evaluation while concurrently addressing relevant psychological comorbidities and physiological factors.

### 4.4 Association of symptom clusters, core symptoms, and survival at one month postoperatively

This study found no significant association between symptom clusters/core symptoms and 1-year postoperative survival. This null finding may reflect contemporary improvements in esophageal cancer outcomes: A recent Swedish population-based study (42) including 2,291 patients who underwent esophagectomy between 2000 and 2020 demonstrated substantial improvements in survival over time. Comparing the most recent surgery period (2015–2020) with the earliest (2000–2004), the adjusted hazard ratio for 5-year all-cause mortality was 0.57 (95% CI 0.47–0.69), with similar trends observed for 1-year all-cause mortality. With increasing centralization of surgical care and optimized perioperative management, short-term mortality at high-volume centers has declined considerably. Notably, fatigue—a pivotal core symptom with bridge functionality in our network analysis—is established as a key determinant of impaired quality of life (43). Thus, in an era of improved survival, precision management targeting fatigue represents a critical strategy for optimizing quality of life, distinct from survival-focused interventions.

### 4.5 Advantages and limitations

The principal strengths of this study include its prospective design, high participation rate, and application of a well-validated questionnaire. To our knowledge, while similar studies exist, few specifically target the critical one-month postoperative phase while incorporating esophageal cancer-specific symptoms into network analysis and examining their association with 1-year survival.

Nevertheless, these findings warrant cautious interpretation. Several limitations should be acknowledged. First, this was a single-center study conducted at a tertiary hospital in China, which may limit the generalizability of our findings to other populations or healthcare settings. Second, the cohort was predominantly male (96.7%), reflecting the higher incidence of esophageal cancer in men, but this extreme sex imbalance limits the applicability of our findings to female patients. Third, the cohort composition may have been subject to selection bias, as only patients who survived to the 1-month postoperative landmark and completed symptom assessment were included. Fourth, symptom clusters were defined as present if ≥50.0% of participants reported constituent symptoms. However, this conventional threshold for cluster classification may inherently influence outcomes. Fifth, patient-reported symptoms were dichotomized: ratings of 3 (“quite a bit”) or 4 (“very much”) denoted presence, while 1 (“not at all”) or 2 (“a little”) indicated absence. Although this ≥3 cutoff for symptom presence aligns with prior cancer symptom cluster studies, it may underestimate subclinical burden by excluding patients scoring 2 who may still require clinical attention. Finally, symptom cluster definitions may exhibit time-dependency. Critically, the contemporaneous symptom networks derived from this cross-sectional analysis cannot establish causal relationships between symptoms at 1-month post-esophagectomy.

Therefore, longitudinal studies with larger, more diverse, and sex-balanced cohorts are recommended to validate our findings and improve generalizability. Such research would delineate the temporal evolution of postoperative symptoms in esophageal cancer patients and provide stronger evidence for inferring symptom causality, thereby informing comprehensive management strategies.

## 5 Conclusion

This study elucidates the multidimensional symptom network among 361 patients at 1 month post-esophagectomy, and prospectively examines the associations between symptom clusters/core symptoms and 1-year postoperative survival using a 1-month landmark design in a longitudinal cohort.

Critically, although no statistically significant association between early postoperative symptoms and 1-year survival was detected in this cohort, fatigue significantly impairs patients’ quality of life. This reframes clinical priorities from solely extending survival to improving quality of life, representing an important shift in cancer care philosophy.

Our findings suggest that symptom network centrality metrics may serve as potentially useful research endpoints in oncology practice. Routine clinical data can be applied to quantify symptom severity, construct symptom networks, and calculate centrality indices to guide evidence-based symptom management. Future prospective studies are needed to validate whether centrality-based targeting translates into improved patient-reported outcomes. Future studies should develop time-resolved dynamic symptom networks to track centrality trajectories, clarify causal interrelationships between symptoms, and identify actionable therapeutic targets. The long-term prognostic value of postoperative symptom clusters warrants further investigation in larger cohorts with extended follow-up.

## Data Availability

The raw data supporting the conclusions of this article will be made available by the authors, without undue reservation.
