# Supplementary material for: Symptom clusters and core symptoms in early postoperative esophageal cancer patients
Source: Front Gastroenterol (Lausanne). 2026 Sep 22;5:1766306. doi: 10.3389/fgstr.2026.1766306 (PMC13638374; doi:10.3389/fgstr.2026.1766306)
Supplement: Supplementary file 1 [file DataSheet1.doc]

Supplementary Material

# 1 Supplementary Table

# Table S1 Occurrence of symptoms and results of symptom cluster analysis (n=361)

| Symptoms | Number of cases (%) | Factor analysis | | | | |
| --- | --- | --- | --- | --- | --- | --- |
|  |  | Factor1 | Factor 2 | Factor 3 | Factor 4 | Factor 5 |
| Eating | 206（57.1） | **.68** | .03 | .20 | .18 | -.09 |
| Reflux | 168（46.5） | **.61** | .16 | .21 | -.10 | .24 |
| Choking  when swallowing | 134（37.1） | **.61** | .02 | .30 | -.29 | -.09 |
| Appetite loss | 128（35.5） | **.55** | .09 | .13 | .25 | -.11 |
| Nausea and vomiting | 100（27.7） | **.55** | .06 | -.35 | .26 | .30 |
| Dysphagia | 336（93.1） | **-.45** | .43 | -.09 | .24 | -.07 |
| Cough | 136（37.7） | -.03 | **.70** | -.01 | -.10 | .04 |
| Dyspnoea | 93（25.8） | .16 | **.66** | .14 | .03 | .04 |
| Insomnia | 77（21.3） | .11 | **.63** | .10 | .24 | -.13 |
| Esophageal pain | 99（27.4） | .16 | .02 | **.77** | .11 | .15 |
| Pain | 141（39.1） | .03 | .19 | **.75** | .22 | .02 |
| Fatigue | 270（74.8） | .18 | .33 | .29 | **.62** | .11 |
| Dry mouth | 109（30.2） | .08 | -.01 | .03 | **.62** | .03 |
| Trouble talking | 143（39.6） | -.07 | .02 | .11 | **.62** | -.06 |
| Constipation | 44（12.2） | .02 | .05 | .06 | .61 | **-.74** |
| Diarrhea | 79（21.9） | .03 | .02 | .23 | .13 | **.69** |

| **Total Variance Explained** | | | | | | | | | |
| --- | --- | --- | --- | --- | --- | --- | --- | --- | --- |
| Component | Initial Eigenvalues | | | Extraction Sums of Squared Loadings | | | Rotation Sums of Squared Loadings | | |
|  | Total | % of Variance | Cumulative % | Total | % of Variance | Cumulative % | Total | % of Variance | Cumulative % |
| 1 | 2.879 | 17.995 | 17.995 | 2.879 | 17.995 | 17.995 | 2.126 | 13.286 | 13.286 |
| 2 | 1.849 | 11.559 | 29.554 | 1.849 | 11.559 | 29.554 | 1.677 | 10.482 | 23.768 |
| 3 | 1.306 | 8.162 | 37.716 | 1.306 | 8.162 | 37.716 | 1.665 | 10.408 | 34.176 |
| 4 | 1.226 | 7.663 | 45.379 | 1.226 | 7.663 | 45.379 | 1.623 | 10.144 | 44.320 |
| 5 | 1.097 | 6.856 | 52.235 | 1.097 | 6.856 | 52.235 | 1.266 | 7.915 | 52.235 |
| 6 | .976 | 6.101 | 58.336 |  |  |  |  |  |  |
| 7 | .944 | 5.897 | 64.234 |  |  |  |  |  |  |
| 8 | .818 | 5.115 | 69.349 |  |  |  |  |  |  |
| 9 | .805 | 5.032 | 74.381 |  |  |  |  |  |  |
| 10 | .760 | 4.748 | 79.129 |  |  |  |  |  |  |
| 11 | .713 | 4.454 | 83.583 |  |  |  |  |  |  |
| 12 | .626 | 3.911 | 87.494 |  |  |  |  |  |  |
| 13 | .586 | 3.665 | 91.158 |  |  |  |  |  |  |
| 14 | .513 | 3.203 | 94.362 |  |  |  |  |  |  |
| 15 | .457 | 2.859 | 97.221 |  |  |  |  |  |  |
| 16 | .445 | 2.779 | 100.000 |  |  |  |  |  |  |
| Extraction method: principal component analysis. | | | | | | | | | |

**Table S2 Results of multifactor logistic regression analysis**

| Symptom cluster | Variable | Odds Ratio | 95% CI | P Value |
| --- | --- | --- | --- | --- |
| Eating-related | Male (reference) | - | - |  |
|  | Female | 7.06 | 1.24-1.33 | 0.069 |
|  | ＜60（reference） | - | - |  |
|  | 60-74 | 1.14 | 0.70-1.84 | 0.605 |
|  | ≥75 | 1.37 | 0.26-7.69 | 0.707 |
|  | 0-Ⅰ（reference） | - | - |  |
|  | II | 1.58 | 0.83-3.05 | 0.170 |
|  | III-IV | 1.09 | 0.64-1.87 | 0.739 |
|  | Squamous cell carcinoma（reference） |  |  |  |
|  | Adenocarcinoma | 1.23 | 0.67-1.28 | 0.765 |
|  | Ivor Lewis（reference） | - | - |  |
|  | McKeown | 0.91 | 0.54-1.52 | 0.714 |
|  | Neoadjuvant therapy | 1.40 | 0.85-2.32 | 0.184 |
|  | Postoperative complications | 0.87 | 0.26-2.81 | 0.820 |
|  | Comorbidities | 1.26 | 0.47-3.52 | 0.645 |
| Breathing-related | Male（reference） | - | - | 0.301 |
|  | Female | 2.15 | 0.53-10.65 |  |
|  | ＜60（reference） | - | - |  |
|  | 60-74 | 1.56 | 0.97-2.53 | 0.070 |
|  | ≥75 | 3.86 | 0.76-28.43 | 0.123 |
|  | 0-Ⅰ（reference） | - | - |  |
|  | II | 1.15 | 0.60-2.20 | 0.675 |
|  | III-IV | 1.20 | 0.70-2.05 | 0.500 |
|  | Squamous cell carcinoma（reference） | - | - |  |
|  | Adenocarcinoma | 1.03 | 0.62-1.73 | 0.896 |
|  | Ivor Lewis（reference） | 1.15 | 0.39-3.39 | 0.796 |
|  | McKeown | 0.00 | NA |  |
|  | Neoadjuvant therapy | 1.08 | 0.65-1.77 | 0.774 |
|  | Postoperative complications | 1.74 | 0.54-5.77 | 0.352 |
|  | Comorbidities | 0.86 | 0.31-2.32 | 0.764 |
| Pain-related | Male（reference） | - | - |  |
|  | Female | 1.47 | 0.36-6.64 | 0.593 |
|  | ＜60（reference） |  |  |  |
|  | 60-74 | 0.78 | 0.49-1.26 | 0.318 |
|  | ≥75 | 0.13 | 0.01-0.79 | 0.062 |
|  | 0-Ⅰ（reference） | - | - |  |
|  | II | 0.97 | 0.51-1.85 | 0.923 |
|  | III-IV | 0.88 | 0.52-1.49 | 0.637 |
|  | Squamous cell carcinoma（reference） |  |  |  |
|  | Adenocarcinoma | 0.84 | 0.50-1.40 | 0.506 |
|  | Ivor Lewis（reference） | 1.01 | 0.34-2.97 | 0.986 |
|  | McKeown | 1.94 | 0.18-43.41 | 0.596 |
|  | Neoadjuvant therapy | 1.00 | 0.61-1.64 | 0.995 |
|  | Postoperative complications | 0.60 | 0.18-1.92 | 0.393 |
|  | Comorbidities | 1.06 | 0.40-2.90 | 0.902 |
| Fatigue-speech related | Male（reference） | - | - |  |
|  | Female | 1.93 | 0.00-NA | 0.983 |
|  | ＜60（reference） | - | - |  |
|  | 60-74 | 0.68 | 0.42-1.10 | 0.115 |
|  | age≥75 | 0.35 | 0.05-1.90 | 0.239 |
|  | 0-Ⅰ（reference） | -- |  |  |
|  | II | 0.99 | 0.52-1.92 | 0.987 |
|  | III-IV | 1.07 | 0.62-1.82 | 0.816 |
|  | Squamous cell carcinoma（reference） | - | - |  |
|  | Adenocarcinoma | 0.90 | 0.54-1.52 | 0.703 |
|  | Ivor Lewis（reference） | 1.01 | 0.35-2.97 | 0.981 |
|  | McKeown | 2.05 | 0.19-45.80 | 0.567 |
|  | Neoadjuvant therapy | 0.75 | 0.45-1.23 | 0.253 |
|  | Postoperative complications | 1.04 | 0.33-3.36 | 0.941 |
|  | Comorbidities | 0.89 | 0.33-2.37 | 0.819 |
| Excretory-related | Male（reference） | - | - |  |
|  | Female | 2.21 | 0.55-10.96 | 0.282 |
|  | ＜60（reference） | - | - |  |
|  | 60-74 | 1.13 | 0.70-1.83 | 0.604 |
|  | ≥75 | 2.61 | 0.52-19.15 | 0.271 |
|  | 0-Ⅰ（reference） | -- |  |  |
|  | II | 1.39 | 0.73-2.67 | 0.315 |
|  | III-IV | 1.19 | 0.70-2.03 | 0.511 |
|  | Squamous cell carcinoma（reference） | - | - |  |
|  | Adenocarcinoma | 1.10 | 0.66-1.84 | 0.706 |
|  | Ivor Lewis（reference） |  |  |  |
|  | McKeown | 0.54 | 0.02-6.01 | 0.627 |
|  | Neoadjuvant therapy | 0.73 | 0.45-1.20 | 0.220 |
|  | Postoperative complications | 1.30 | 0.40-4.21 | 0.659 |
|  | Comorbidities | 0.85 | 0.31-2.27 | 0.747 |
| Fatigue | Male（reference） | - | - |  |
|  | Female | 2.11 | 0.35-40.61 | 0.494 |
|  | ＜60（reference） | - | - |  |
|  | 60-74 | 1.84 | 1.06-3.23 | **0.032** |
|  | ≥75 | 3.16 | 0.48-62.29 | 0.304 |
|  | 0-Ⅰ（reference） | - | - |  |
|  | II | 1.01 | 0.50-2.10 | 0.970 |
|  | III-IV | 1.79 | 0.95-3.48 | 0.079 |
|  | Squamous cell carcinoma（reference） | - | - |  |
|  | Adenocarcinoma | 0.84 | 0.46-1.53 | 0.559 |
|  | Ivor Lewis（reference） |  |  |  |
|  | McKeown | 0.68 | 0.06-15.26 | 0.758 |
|  | Neoadjuvant therapy | 1.86 | 1.05-3.35 | **0.036** |
|  | Postoperative complications | 0.97 | 0.28-3.46 | 0.962 |
|  | Comorbidities | 0.68 | 0.24-2.07 | 0.475 |
| Pain | Male（reference） | - | - |  |
|  | Female | 0.96 | 0.22-3.83 | 0.952 |
|  | ＜60（reference） | - | - |  |
|  | 60-74 | 0.89 | 0.55-1.45 | 0.648 |
|  | ≥75 | 1.92 | 0.39-10.44 | 0.417 |
|  | 0-Ⅰ（reference） | - | - |  |
|  | II | 1.72 | 0.90-3.29 | 0.101 |
|  | III-IV | 1.00 | 0.58-1.72 | 0.992 |
|  | Squamous cell carcinoma（reference） | - | - |  |
|  | Adenocarcinoma | 0.83 | 0.49-1.40 | 0.493 |
|  | Ivor Lewis（reference） |  |  |  |
|  | McKeown | 0.86 | 0.04-9.56 | 0.906 |
|  | Neoadjuvant therapy | 1.15 | 0.70-1.91 | 0.575 |
|  | Postoperative complications | 0.82 | 0.24-2.67 | 0.742 |
|  | Comorbidities | 0.98 | 0.36-2.64 | 0.973 |
| Insomnia | Male（reference） | - | - |  |
|  | Female | 1.86 | 0.37-7.74 | 0.406 |
|  | ＜60（reference） | - | - |  |
|  | 60-74 | 0.98 | 0.54-1.78 | 0.949 |
|  | ≥75 | 1.35 | 0.18-7.03 | 0.734 |
|  | 0-Ⅰ（reference） | - | - |  |
|  | II | 1.50 | 0.71-3.11 | 0.276 |
|  | III-IV | 0.69 | 0.33-1.37 | 0.292 |
|  | Squamous cell carcinoma（reference） | - | - |  |
|  | Adenocarcinoma | 1.03 | 0.54-1.92 | 0.938 |
|  | Ivor Lewis（reference） |  |  |  |
|  | McKeown | 1.03 | NA | 0.986 |
|  | Neoadjuvant therapy | 0.78 | 0.42-1.44 | 0.424 |
|  | Postoperative complications | 0.50 | 0.09-2.34 | 0.399 |
|  | Comorbidities | 0.99 | 0.26-3.14 | 0.989 |

# 2 Supplementary Figures

**Figure.S1 Centrality measure**

**Figure.S2 Bridge Centrality measures**

# 3 QOL questionnaires
